# Editorial: Neural interfaces for sensory input

**DOI:** 10.3389/fnins.2024.1515353

**Published:** 2024-11-22

**Authors:** Aaron M. Dingle, Elizabeth B. Torres, Jason A. Brant, Dayo O. Adewole

**Affiliations:** ^1^Department of Surgery, Division of Plastic Surgery, University of Wisconsin-Madison, Madison, WI, United States; ^2^Graduate School, Rutgers University, Newark, NJ, United States; ^3^Department of Surgery, Division of Otolaryngology, University of Wisconsin-Madison, Madison, WI, United States; ^4^University of Pennsylvania, Philadelphia, PA, United States

**Keywords:** neural interface, sensory input, neuroprostheses, medical devices, neural interface design

Sensory neural interfaces are devices capable of detecting, transducing and delivering sensory inputs to either the central or peripheral nervous system. The past decade has seen a surge in neural interface research seeking to develop novel systems that restore sensory functions—from advancing the current state of hearing restoration, to peripheral and central interfaces to restore the sense of touch. These devices now stretch the full gamut of invasive to non-invasive, and primarily utilize electrical stimulation of the nervous system, although alternative methods such as optogenetics have also increased in prevalence. The collection of articles on the current Research Topic identifies key aspects at the forefront of restoring sensory function, providing examples and guidance toward delivering state-of-the-art sensory prosthetic technologies for a range of clinical applications.

Cochlear implants (CIs) to restore hearing represent the most prevalent and recognizable sensory neural interface in modern society. With four decades of clinical success restoring hearing for hundreds of thousands of individuals, even the most modern iterations of these implants have their limitations. Zamaninezhad et al. investigated the highly variable performance of CIs among users, with a focus on speech perception. Cochlear health—defined as the number and extent of the functionality of spinal ganglion neurons—is understood to impact speech intelligibility in CI users, but is not readily testable in the clinical setting. Unlike previous studies of cochlear health which have relied on histological methodologies, this work sought to identify clinically applicable cochlear health measures by investigating the effects of (1) stimulation polarity and (2) the relative contribution of different frequency bands on the speech perception of living CI users. The consenting participants were 13 bilateral CI users, with a mean age of 58 years. The study demonstrated significant correlations between increased interphase gap (IPGE_slope_) and speech perception, and IPGE_slope_ and age in humans, thus highlighting IPGE_slope_ as a potential clinical measure of cochlear health. More broadly, in order to advance the field of hearing restoration, it is essential to understand auditory signal processing and the current technological limitations to clinical problems. In their review, Quimby et al. succinctly introduce the essential anatomical pathways and biological mechanisms responsible for hearing, from the cochlea to the auditory cortex. With the essential neural anatomy and mechanisms laid out, this review goes on to cover the current state-of-the-art of cochlear implants and promising emerging technologies (e.g., optogenetics), as well as covering current and future limitations to advancing the state of hearing restoration.

Prosthetic devices to replace lost limbs have benefited substantially from advances in neuroprosthetics over the past decade, with consequent increased public support. Restoration of a sense of touch represents the current pinnacle of upper- and lower-limb neuroprosthetics, allowing for true closed-loop control, improved balance with lower-limb prosthetics, and other functional benefits. Li et al. investigated whether the sensation of touch elicited via a neural interface interfered with essential reflexes for sensorimotor control (i.e., the H-reflex) in persons with lower limb loss. The consenting participants were two males over 50 years old who had both experienced transtibial amputation 9 years prior. Both participants had previously received composite flat interface nerve cuff electrodes (C-FINE), allowing electrically elicited plantar sensation and H-reflex activation of the missing limb in various postures. Interestingly, the results of this study were in opposition to the original hypothesis, demonstrating that elicited plantar sensation did not alter participants' H-reflex under the experimental conditions. Ng et al. explored novel ways for neural prostheses to encode and deliver sensory inputs. This work demonstrated that both frequency and intensity of touch could be semi-independently encoded within a single electrical simulation channel. Rather than varying current magnitude to encode intensity (which can cause pain at higher intensities) or varying stimulation rate to encode frequency, this new approach uses pulses of a fixed current magnitude. By varying the amount of time between bursts of stimulation pulses (to modulate frequency) or varying the number of pulses within each burst (to modulate intensity), these researchers developed a multiplexed encoding scheme that only requires 1 channel for stimulation, and were able to elicit varying intensities across several frequencies in able-bodied human subjects. Raghav Hari Krishna et al. explored the implications of electrotactile feedback on balance in ten (consenting) young (average age of 24.7 years), healthy human subjects. Non-invasive transcutaneous stimulation of the medial calcaneal nerve was applied to augment plantar cutaneous feedback while participants performed simultaneous balancing and cognitive tasks. The data demonstrates electrotactile feedback improves balance and that balance is improved further when the stimulation is proportional to the task. This research identifies several fascinating paradigms associated with balance in healthy individuals; such as the impact of oscillation frequency and balance time, while also identifying numerous gaps in knowledge and methodology required to advance the field of peripheral sensory augmentation to address balance issues.

Key components in the clinical translation of sensory neuroprosthetics are their design/development and pre-clinical efficacy. From the design and development perspective, Thota and Jung provide a valuable overview of the common processes utilized by neurotechnology developers to bring novel medical devices to market and ultimately deliver lifesaving and life sustaining clinical care. This article eloquently explains the commonly accepted Waterfall methodology utilized by established medical device manufacturers, and proposes the application of the Agile methodology as a means to accelerate the clinical translation of neurotechnologies. The limitations of both methodologies are discussed, utilizing peripheral nerve interface development to illustrate the pros and cons of both methodologies alongside examples of hybrid methodologies as a means to improving delivery of clinical devices. From the pre-clinical efficacy perspective, Smith et al. describe a novel methodology for studying intracortical microstimulation (ICMS) of the somatosensory cortex in rats. This model utilizes operant conditioning behavioral tasks to assess ICMS-evoked sensory perception thresholds, representing a readily accessible and ethically preferred alternative to the more common non-human primate models. By modifying a well-established nose-poke auditory task paired with microelectrode arrays (MEAs) implanted to target the forelimb area of the left primary somatosensory cortex (S1FL), this model presents a highly accurate behavioral paradigm to access ICMS-evoked somatosensory perception thresholds. This novel, validated methodology represents a new tool for evaluating MEAs for future clinical applications.

The complexity of the human sensory experience has motivated the development of several different approaches to restore sensory function. This Research Topic provides a range of both clinical and research-driven perspectives on sensory restoration. As the field of sensory neural interfaces evolves, we expect these perspectives will serve as the foundation for future insights into improved clinical outcomes for prosthesis users and better quality of life for those living with sensory disabilities.

